# Spontaneous Retroperitoneal Hemorrhage in a Patient with Prolymphocytic Transformation of Chronic Lymphocytic Leukemia

**DOI:** 10.1155/2013/802376

**Published:** 2013-08-19

**Authors:** Gwynivere A. Davies, Alejandro Lazo-Langner, Michael Shkrum, Leonard Minuk

**Affiliations:** ^1^Department of Medicine, Western University, London, ON, Canada; ^2^Division of Hematology, Department of Medicine, Western University, London Health Sciences Centre, Room E6-219, Victoria Hospital, 800 Commissioners Road East, P.O. Box 5010, London, ON, Canada N6A 4G5; ^3^Department of Epidemiology and Biostatistics, Western University, London, ON, Canada; ^4^Department of Pathology, Western University, London, ON, Canada

## Abstract

Prolymphocytic transformation of chronic lymphocytic leukemia is a rare but recognized entity. We present the case of a 76-year-old gentleman with a previous diagnosis of chronic lymphocytic leukemia who presented with fatigue, fever, and a white blood cell count of 500 000 with prolymphocytes on peripheral blood examination. Chlorambucil and dexamethasone were initiated. He developed progressive anemia during his admission with no clear cause on initial CT examination. Bilateral hip pain began several days later and he was unfortunately diagnosed with a large spontaneous retroperitoneal hemorrhage postmortem. This condition is rare and generally occurs in those receiving therapeutic anticoagulation or dialysis, with known bleeding disorders or vascular malformation, none of which were present in our patient. Pathology revealed marked leukemoid engorgement of the vessels of many organs with prolymphocytes. We discuss the potential etiologies and relationships between these critical diagnoses.

## 1. Introduction

Chronic lymphocytic leukemia (CLL) is a hematologic disorder characterized by overproduction of circulating B cells [1]. These lymphocytes are small, mature cells that are clonal in nature, and express a characteristic flow cytometric pattern. CLL is generally a slowly progressive disease that is monitored without therapy until symptoms develop; however Richter's transformation to aggressive histology lymphoma can occur. More rarely, transformation to Hodgkin's disease, lymphoblastic lymphoma, small noncleaved cell lymphoma, hairy cell leukemia, T-cell leukemia, and prolymphocytic leukemia (PLL) has been reported [2].

## 2. Case Presentation

We describe the case of a 76-year-old gentleman diagnosed with Rai stage 0 CLL 10 months prior who presented with fatigue and fever. Past medical history included nephrolithiasis, repaired right hydrocele, hypertension, osteoarthritis, remote gastric ulcer, and prostate adenocarcinoma for which he received 35 fractions of external beam radiation in early 2011. In addition, the patient worked as a farmer and had frequent exposure to various pesticides and herbicides. During urologic followup in September 2011 he was found to have a mild thrombocytopenia and asymptomatic lymphocytosis of 20 000 × 10^6^/L. Flow cytometry demonstrated 91% lymphocytes with a monoclonal B-cell population composing 85% of total leukocytes that were CD19/CD5, CD20, CD23, and FMC7 positive expressing dim kappa light chains; CD38 was positive on 90% of CD19/CD5+ cells. FISH showed a typical CLL deletion at 13q14.3 only.

**Figure 1:**
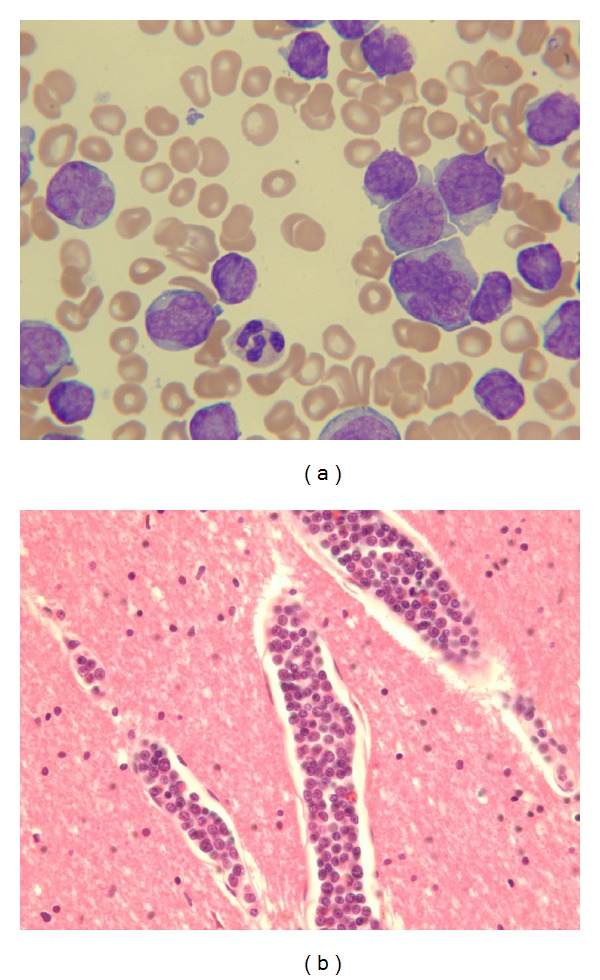
(a) Peripheral blood film demonstrating prolymphocytic population. (b) Representative vessels engorged by leukemic cells. Cerebrum, H&E stain, 400×.

During subsequent visits, splenomegaly was detected, but he was otherwise well with no infectious or constitutional symptoms until he presented acutely unwell in June 2012. Investigations revealed anemia with a hemoglobin (Hgb) of 7.6 g/dL, platelets of 40 000 × 10^6^/L, and a leukocyte count of approximately 50 0000 × 10^6^/L. Differential showed 278 000 × 10^6^/L immature lymphocytes and 199 000 × 10^6^/L lymphocytes, consistent with a transformation to PLL. Pleomorphic abnormal large lymphoid cells with 1+ nucleoli (Figure 1(a)) were seen on peripheral smear. Results also showed elevation of liver enzymes and LDH. The patient was started on dexamethasone and chlorambucil as cytoreductive chemotherapy. In addition, he received levofloxacin for pneumonia and was transfused 2 units of packed cells as his Hgb decreased to 6.2 g/dL on day 1 after admission. Given the patient's anemia and aggressive hematologic presentation, he had a CT of his chest, abdomen, and pelvis performed to rule out Richter's syndrome; this showed splenomegaly and an absence of enlarged lymph nodes or bleeding.

Four days after his admission, the patient developed mild confusion and new onset left hip pain. He was evaluated by neurology for bilateral hip flexor weakness who ordered an MRI and conduction studies. The following day his hemoglobin dropped from 8.8 to 6.4 g/dL with platelets of 45 000 × 10^6^/L and leukocytes of 397 000 × 10^6^/L. The patient was again transfused 2 units of packed cells. Coagulation studies showed an elevated INR of 1.4, with normal PTT of 32, arguing against a diagnosis of disseminated intravascular coagulation (DIC). Fibrinogen was not performed. Later that evening, he developed contralateral burning hip pain requiring increasing analgesics, as well as intermittent nausea with several episodes of emesis. Vital signs were stable with the exception of mild hypotension with a blood pressure of 90/61 early the next morning; however the patient was found unresponsive 1 hour later. Full resuscitation efforts were attempted but unfortunately the patient died of a pulseless electrical activity (PEA) arrest. An autopsy was performed and examination showed marked leukemoid engorgement in the vessels of many organs (Figure 1(b)). Leukemic infiltrates were seen in the spleen and bone marrow. A 20 × 13 × 5 cm right retroperitoneal hemorrhage was identified with no apparent source. Serosal hemorrhage in the cecum and ascending colon, mesenteric hemorrhages, and a small hemoperitoneum were noted.

## 3. Discussion

CLL is associated with phenomena such as autoimmune hemolytic anemia and thrombocytopenia [1]. On review of the literature there are two previous reports of spontaneous hematoma with this disease process. The first occurred in a patient with known CLL and the other was subsequently diagnosed two months after the bleed. In both cases, an idiopathic factor VIII inhibitor was identified as the likely cause [3, 4]. Other potential etiologies for spontaneous bleeding in CLL include thrombocytopenia secondary to marrow infiltration or consumption, acquired von Willebrand's disease [5], acquired platelet defects potentially secondary to abnormal platelet factor-3 availability and abnormal aggregation [6] and vessel fragility secondary to intimal infiltration as is seen in systemic amyloidosis [7].

This case is unique from previous reports due to the parallel presentation of transformation to PLL and spontaneous retroperitoneal hemorrhage (SRH), two rare conditions not previously associated. PLL is an acute process diagnosed when the prolymphocyte count exceeds 55% of lymphoid cells in the peripheral blood [8]. In contrast to Richter's syndrome, there is usually a lack of lymphadenopathy or extranodal infiltration though massive splenomegaly is common. SRH is defined in the absence of recent procedures or operations, trauma, or abdominal aortic aneurysms [9] and is mainly seen in patients on therapeutic anticoagulation, hemodialysis, or with bleeding disorders [10]. Sunga et al. (2012) [9] performed an 8-year observational cohort and identified 89 SRHs of which only 15.3% were not on antiplatelet or anticoagulation agents or both. This is concerning for an increased incidence as current standard practice for inpatients now usually includes prophylactic low-dose anticoagulation to prevent thrombosis, even in patients with mild to moderate cytopenias.

Disease processes such as amyloidosis, which results in abnormal deposits in vessel walls, can lead to widespread fragility and predispose to bleeding [7]. Torres et al. [11] also suggested that arteriosclerotic deposits can cause vessel fragility leading to retroperitoneal iliopsoas hemorrhage; this matches the finding that small nonaortic aneurysms were the most common cause of SRH in the previously described cohort [9]. In contrast, our patient had engorgement of his vessels with prolymphocytes, but no actual intramural invasion or aneurysm identified. Leukocytic infiltrates have previously been postulated to lead to inflammation related thrombosis [12], and certainly the number of cells present would be concerning for sudden venous congestion and rupture, or even a bleeding diathesis as in hyperviscosity syndrome, not previously reported in PLL.

Mortality for SRH is high, ranging between 12% and 19.1% [9, 13]. Misdiagnosis occurs in approximately 10.1% of cases as symptoms of abdominal pain, hip, and upper thigh pain can be nonspecific. When associated with acute muscle weakness, the presentation suggests a more peripheral etiology and can lead to extremity rather than central imaging. Once identified, SRH management includes supportive therapy with volume replacement, blood products, and reversal of anticoagulants. Severe, life threatening SRH may also require percutaneous endovascular therapy or open surgical exploration [14].

In summary, we present a case of prolymphocytic transformation of CLL associated with spontaneous haemorrhage into the retroperitoneal cavity, which was unfortunately identified postmortem. The most likely etiology for this SRH was leukemic vascular congestion with thrombosis and rupture, but other contributing factors likely included thrombocytopenia, ineffective coagulation secondary to hyperviscosity and use of prophylactic dose low molecular weight heparin. Despite the cytopenias frequently seen in aggressive hematologic processes such as PLL, physicians should have a high clinical suspicion for this entity with any sudden drop in hemoglobin associated with symptoms of new abdominal, back, or hip pain.
